# GSDMD-mediated pyroptosis restrains intracellular *Chlamydia trachomatis* growth in macrophages

**DOI:** 10.3389/fcimb.2023.1116335

**Published:** 2023-03-16

**Authors:** Ping Jiang, Hongzhi Chen, Xiaojing Feng, Huiqi Xie, Mengjie Jiang, Danning Xu, Haoneng Tang, Ningjie Zhang, Jianlin Chen, Lei Zhang, Lingli Tang

**Affiliations:** ^1^ Department of Laboratory Medicine, The Second Xiangya Hospital, Central South University, Changsha, Hunan, China; ^2^ National Clinical Research Center for Metabolic Disease, Key Laboratory of Diabetes Immunology, Ministry of Education, Metabolic Syndrome Research Center, and Department of Metabolism and Endocrinology, The Second Xiangya Hospital, Central South University, Changsha, Hunan, China; ^3^ Department of Blood Transfusion, The Second Xiangya Hospital, Central South University, Changsha, Hunan, China; ^4^ Reproductive Medicine Center, Department of Obstetrics and Gynecology, The Second Xiangya Hospital, Central South University, Changsha, Hunan, China; ^5^ Department of Urology, The Second Xiangya Hospital, Central South University, Changsha, Hunan, China

**Keywords:** *Chlamydia trachomatis*, GSDMD, pyroptosis, macrophage, caspase-1, caspase-11

## Abstract

Pyroptosis, a type of programmed necrosis associated with inflammatory, is a host defense mechanism against microbial infections. Although Chlamydia has been shown to induce pyroptosis, whether pyroptosis directly impacts the growth of Chlamydia has not been demonstrated. In this study, we found that *C. trachomatis* L2 infection of the mouse macrophage RAW 264.7 cells induced pyroptosis by monitoring the ultrastructural changes under transmission electron microscopy and the release of LDH and IL-1β. More importantly, this *C. trachomatis*-triggered pyroptosis with activation of caspase-1 and caspase-11 was also accompanied by gasdermin D (GSDMD) activation. Suppression of these two inflammatory caspases inhibited GSDMD activation. Interestingly, the *C. trachomatis*-triggered pyroptosis significantly inhibited the intracellular growth of *C. trachomatis* since inactivation of either GSDMD or caspase-1/11 significantly rescued infectious *C. trachomatis* yields, which suggests pyroptosis response can be utilized as an intrinsic mechanism to restrict *C. trachomatis* intracellular infection in addition to the well- documented extrinsic mechanisms by recruiting and enhancing inflammatory responses. This study may reveal novel targets for attenuating *C. trachomatis* infectivity and/or pathogenicity.

## 1 Introduction

*Chlamydia trachomatis* (*C. trachomatis*), an obligate intracellular Gram-negative bacterium, is a major pathogen of human sexually transmitted disease increasing public health burden (Taylor-Robinson, 2017). *C. trachomatis* has a unique biphasic development cycle. Initially, the infectious, extracellular elementary body (EB) infects the epithelial host cell. Intracellularly, the EB transforms to the metabolically active reticulate body (RB) for replication. The progeny RBs condense into EBs, which will be released when the host cell breaks down. At this time, macrophages recruited to sites of tissue infection can internalize *C. trachomatis* by phagocytosis (Morrison and Morrison, 2000). However, the intracellular fate of *C. trachomatis* in macrophages is still controversial. Some studies have found that *C. trachomatis* can survive in monocytes and macrophages for more than 7 days, which facilitates persistent *C. trachomatis* infection (Zuck et al., 2017; Lausen et al., 2019; Sumiyoshi et al., 2019). It has also been found that reactive arthritis caused by *C. trachomatis* infection in the urogenital tract is likely to be caused by the spread of monocyte-macrophages carrying active *C. trachomatis* through the circulatory system (Sumiyoshi et al., 2019). However, a large body of evidence indicated that macrophages can rapidly clear *C. trachomatis* infection to limit *C. trachomatis* intracellular reproduction (Stagg et al., 1998; Qiu et al., 2008; Ziklo et al., 2016). Recently, Faris et al. found that although macrophages support the intracellular growth of *C. trachomatis* serotype L2, they also play a role in resisting *C. trachomatis* infection (Faris et al., 2019). Therefore, understanding the interaction between macrophages and *C. trachomatis* is critical to understand *C. trachomatis* infectivity and pathogenicity.

Pyroptosis, a type of pro-inflammatory programmed cell death, is characterized by cell swelling and cell membrane rupture, and the release of proinflammatory cytokines. Inflammatory caspases (caspase-1/–4/–5/–11) are activated during pyroptosis (Gram et al., 2019). These active caspases cleave gasdermin-D (GSDMD), the effector of pyroptosis, at a link region between the N- and C-terminus, releasing the N-terminus fragment from the inhibitory C-terminal domain. The N-terminus of GSDMD (GSDMD-NT) binds to phosphoinositides located in the inner side of the plasma membrane and forms pores on the membrane leading to cell lysis (Sborgi et al, 2016). Inflammatory cytokine, IL-1 and IL-18 processed by caspase-1, are released during cell lysis or from the pores formed by GSDMD-NT (Shi et al., 2015).

Pyroptosis is an important type of cell death for defending against intracellular pathogen infection. GSDMD plays a key role in mediating the inhibition of Salmonella, Legionella pneumophila and Francisella infections in macrophages (Zhu et al., 2018; Gonçalves et al., 2019; Xia et al., 2019). It has been reported that *C. trachomatis* induces pyroptosis indicated by the releases of LDH and IL-1β *via* activating caspase-1 and caspase-11 in macrophages (Finethy et al., 2015; Webster et al., 2017). However, pyroptosis has not previously been demonstrated to directly impact the growth or development of Chlamydia, although inferring that pyroptosis has negative effects on Chlamydia infection. More direct experimental evidence is needed to confirm the role of pyroptosis in Chlamydia-infected macrophages. This study directly investigate the effect of pyroptosis on chlamydial replication and development and also first investigate the role of GSDMD in Chlamydia infection. We demonstrated that *C. trachomatis* induced GSDMD-mediated pyroptosis *via* activating caspase-1/11 in RAW264.7 cells, which caused cell lysis and pro-inflammatory cytokines release, and subsequent chlamydial clearance.

## 2 Materials and methods

### 2.1 Cells, bacteria, and infections

RAW264.7 cells, HeLa229 cells and iBMDM cells were purchased from the American Type Culture Collection (ATCC) and maintained in DMEM supplemented with 10% FBS. Cells were incubated at 37°C in a humidified incubator of 5% CO_2_, and sub-cultured every 2~3 days. *C. trachomatis* L2/LGV-434/Bu was also obtained from ATCC and was inoculated and propagated in HeLa cells as described previously (Zhong et al., 2001). As for infections, RAW264.7 cells and HeLa cells either grown in tissue culture dishes or plates were inoculated with chlamydial organisms as described previously (Zhong et al., 2001). For inhibitor experiments, we pretreated the cells for 30 minutes with Ac-YVAD-cmk (20 or 50μM), or Necrosulfonamide (5, 10 or 20μM) inhibitors prior to *C. trachomatis* L2 infection, respectively. The same concentrations of the inhibitors were maintained throughout the rest of the infection.

### 2.2 Antibodies and reagents

Primary antibodies against caspase-1 (ab179515), caspase-11 (ab180673), GSDMD (ab209845), GSDME (ab215191) and Biotin Goat polyclonal to *Chlamydia trachomatis* (ab20387) were obtained from Abcam (Cambridge, UK), Antibody for Chlamydia HSP60 (sc57840) was bought from Santa Cruz Biotechnology (California, USA), Antibodies for caspase-8 (4790), caspase-8 p18 (8592), β-Tubulin (2128) and GAPDH (5174) were obtained from Cell Signaling Technology (Danvers, MA, USA), while secondary antibodies (SA00001-15, SA00001-1) used for immunoblotting and Fluorescein (FITC)-conjugated Affinipure Rabbit Anti-Goat IgG(H+L) (SA00003-4) used for immunofluorescence staining were bought from Proteintech (Chicago, USA). IL-1β (CSB-E08054m) and IL-18 (CSB-E04609m) ELISA kits were obtained from CUSABIO (Wuhan, China). Caspase-1 inhibitor Ac-YVAD-cmk (SML0429) was purchased from Sigma (California, USA), Necrosulfonamide (AG-CR1-3705-M005) was bought from Adipogen (Epalinges, Switzerland). Caspase-11 inhibitor Wedelolactone (T3384) was obtained from Target Mol (Boston, MA). All inhibitors were dissolved in dimethyl sulfoxide (DMSO).

### 2.3 Immunoblot analysis

Culture supernatant was harvested to collect dead/floating cells by centrifugation at 12,000 rpm for 10 minutes at 4°C and pooled with adherent cells washing with PBS buffer three times. All cells were lysed on ice to extract protein. Equal amounts of protein lysates (40ug) were separated on SDS-PAGE gels (CWBIO, China) under reducing conditions and subsequently transferred onto nitrocellulose membranes. The caspase-1 antibody (1:1000 dilution), caspase-11 antibody (1:1000 dilution), GSDMD antibody (1:1000 dilution), GSDME antibody (1:1000 dilution), cHSP60 antibody (1:200 dilution), caspase-8 antibody (1:1000 dilution), caspase-8 p18 antibody (1:1000 dilution), β-Tubulin antibody (1:2000 dilution) and GAPDH antibody (1:2000 dilution) were incubated with polyvinylidene difluoride membranes at 4°C overnight. Then we washed the blots with PBS and incubated them with the previous described secondary antibodies. Protein bands were visualized using ECL detection regents (GE Healthcare, USA). Western blot quantification was performed by measuring band intensity with ImageJ freeware (National Institutes of Health, Bethesda, MD, USA). The quantification of cleaved caspases band was normalized first to GAPDH then to the pro-caspase band intensity. The quantification of GSDMD-NT band was normalized first to GAPDH and then to the full-length GSDMD intensity.

### 2.4 Cytokine analysis

RAW264.7 cells were infected with *C. trachomatis* L2 at a multiplicity of infection (MOI) of 5 for 12 hours. Culture supernatant was harvested by low-speed centrifugation to remove cells from the supernatant. After centrifugation the supernatant was directly used for analysis. IL-1β and IL-18 ELISA kits were used to detect the concentrations of IL-1β and IL-18 cytokines in the cell supernatant according to the manufacturer′s instructions.

### 2.5 Cell morphology observation

RAW264.7 cells with 70% confluence in 24-well plates were infected with *C. trachomatis* L2, then cultured in DMEM supplemented with 10% FBS at indicated times. Cells were visualized and photographed at a 40 X magnification using a light microscope.

### 2.6 Transmission electron microscopy (TEM)

RAW264.7 cells were harvested after infecting with *C. trachomatis* L2 at a MOI of 5 for 12 hours and were fixed by Gluta mirror fixing fluid (Solarbio, China) dehydrated with acetone, soaked and buried with pure acetone and encapsulation liquid (1:1) for 12 hours then pure encapsulation liquid for 12 hours. Samples were placed in a 37°C oven overnight, then placed in a 60°C oven for 12-24 hours for curing. Ultrathin sections were performed using an ultra-microtome (Leica Ultracut UCT, Leica, Austria). Sections were using uranium acetate and lead nitrate double staining and photographed with a HT7700 electron microscope.

### 2.7 siRNA transfections

The double-stranded caspase-11 small interfering RNA (siRNA) (5′CCAUUGAUCGGGCAACCUUTT3′) and double-stranded negative control siRNA were purchased from GenePharma (Suzhou, China). RAW264.7 cells were plated to 60%-70% confluence in 60-mm dishes 24 hours before transfection. 200 pmol per dish caspase-11 siRNA, or negative control siRNA was transfected using Advanced DNA RNA Transfection Reagent™ (Zeta Life, USA) following the manufacturer’s instructions. At 48 hours after transfection, the efficiency of the corresponding gene knockdown was confirmed with western blot.

### 2.8 Immunofluorescence microscopy analysis

To detect *C. trachomatis* inclusion, we infected cells with *C. trachomatis* L2 at a MOI of 5, and cultured cells for different times. Then the cells were fixed by 4% paraformaldehyde for 15 minutes and permeabilized with 0.1% (v/v) Triton X-100 for 5 minutes. Then we blocked the cells for 1 hour and incubated the samples with anti-*Chlamydia trachomatis* antibody at 4°C overnight. Fluorescein (FITC)-conjugated Affinipure Rabbit Anti-Goat IgG (H+L) antibody was used as secondary antibody. The nuclei staining was revealed with the Hoechst 33342 (Solarbio, China) solution for 5 minutes. Cells were washed with PBS solution three times for 5 minutes per-time after the primary and secondary antibody incubations. The final treated cells were observed by Zeiss LSM880 microscope and the immunoflurescence signals were processed with ZEN software.

### 2.9 Infectious chlamydial yields quantitation

RAW264.7 cells were infected with *C. trachomatis* L2 at a MOI of 5 for 40 hours under different treatment conditions. RAW264.7 cells then were lysed to collect *C. trachomatis* infectious progenies to re-infect a new culture of HeLa cells that had been grown to equal confluency. Reinfected HeLa cells were fixed at 24 hours post infection, immunostained and immunofluorescence microscopy quantitative analysis of inclusion number relative to nuclei in HeLa cells was used to estimate *C. trachomatis* infectious yields within RAW264.7 cells.

### 2.10 Propidium iodide (PI) staining

RAW264.7 cells were infected with *C. trachomatis* L2 at a MOI of 5 for 12 hours. On each specimen, we removed the media and rinsed it once with PBS buffer. PI solution (Sigma, USA) diluted into full media with the final concentration of 2 μg/ml was prepared and used to incubate the cells at 37°C for 10 minutes. Then we washed the cells once with PBS and fixed them with 4% paraformaldehyde in PBS for 30 minutes. Fluorescent images of cells were obtained with the Zeiss LSM880 microscope, and fluorescent images were deconvolved using Zen software.

### 2.11 LDH cytotoxicity assay

RAW264.7 cells were infected with *C. trachomatis* L2 at a MOI of 5 for 12 hours. LDH release in the culture medium was measured using a fully Automatic Biochemical Analyzer (Hitachi, Japan).

### 2.12 Statistical analysis

All experiments were performed three times independently. Data were expressed as mean ± standard deviation (SD). Statistical analysis was performed using GraphPad Prism7.0 (GraphPad Software Inc., San Diego, CA, USA). For comparison between two groups, *P* values were determined using two-tailed Student’s *t* tests. For multiple-group comparisons, one-way ANOVAs were used to test whether differences among the group means were statistically significant. *P-*values < 0.05 were considered statistically significant.

## 3 Result

### 3.1 *C. trachomatis*-induced pyroptosis is dependent on the activation of GSDMD

**Figure 1:**
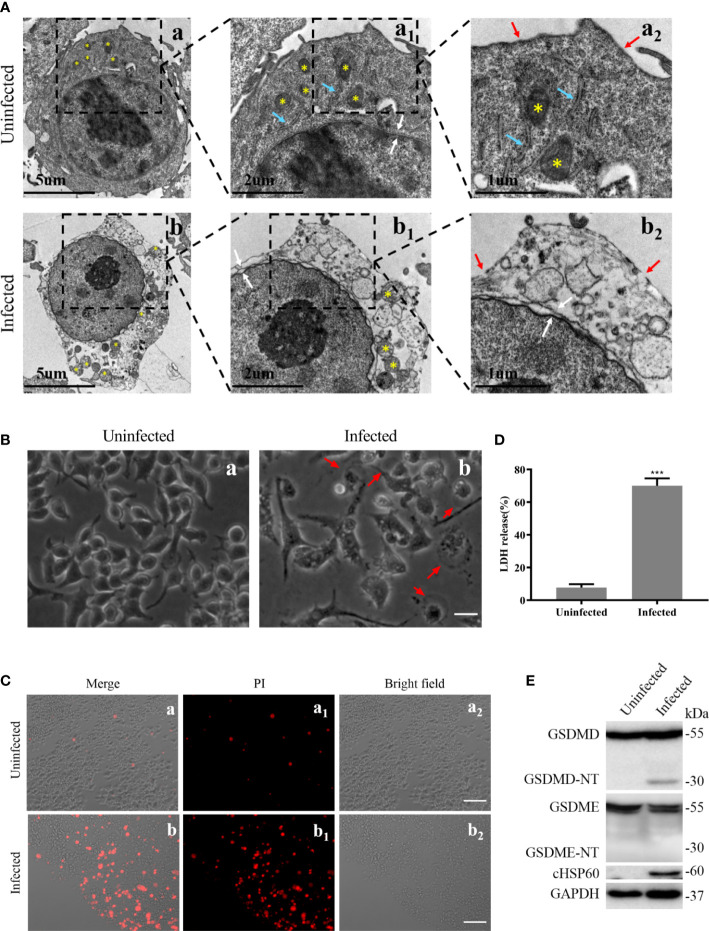
Pyroptosis induced by *C. trachomatis* infection is accompanied by activation of GSDMD in macrophages. RAW264.7 cells were infected with *C*. *trachomatis* (MOI=5) for 12 hours. **(A)**. TEM displayed ultrastructural changes of *C. trachomatis*-infected macrophages. Yellow stars indicated mitochondria, and blue arrowheads represented Golgi, red and white arrowheads represented plasma membrane ruptures and nuclear envelope respectively. **(B)**. Light microscopy revealed signs of pyroptosis in the infected macrophages. Red arrowheads indicate examples of pyroptotic infected cells. Scale bars, 40 μm. **(C)**. PI staining showed increasing membrane permeability of infected RAW 264.7 cells compared with uninfected cells. Scale bars, 20 μm. **(D)**. LDH released in the supernatant was measured to check the release of cellular contents in the RAW 264.7 cells. **(E)**. Immunoblot analysis showed the activation of GSDMD and GSDME in *C. trachomatis-*infected RAW264.7 cells. cHSP60 is as marker of *C. trachomatis* infection. ***P<0.001, vs the uninfected group. n=3.

To determine whether pyroptosis occurs in macrophages during *C. trachomatis* infection, the RAW264.7 macrophages were infected with *C. trachomatis* at a MOI of 5 or left uninfected and multiple pyroptosis indicators were detected at 12 hours post infection. TEM images showed that, compared with uninfected group, RAW264.7 cells infected with *C. trachomatis* had ultrastructural changes including decreased cytoplasmic density, damaged organelles (Mitochondrial swelling and Golgi fragmentation) and fractured cell membrane, but intact nucleus (**Figure 1A**). Light microscopy images displayed that the infected macrophages exhibited typical swelling and characteristic large blebs from the plasma membrane (**Figure 1B**). Moreover, PI staining and LDH release assays were used to estimate cell membrane integrity. We observed a significant increase in PI fluorescence intensity (**Figure 1C**) and LDH release (**Figure 1D**) in the *C. trachomatis*-infected RAW264.7 cells compared to the uninfected cells. Immunoblot analysis detected GSDMD-NT, known as pyroptosis executor, in the RAW264.7 cells at 12 hours post infection (**Figure 1E**). Notably, the N-terminal fragment of cleaved GSDME (GSDME-NT), another member of the gasdermin family which can induce pyroptosis (Wang et al., 2017), was not detected (**Figure 1E**). Taken together, these results strongly demonstrated that *C. trachomatis* infection induced GSDMD-mediated pyroptosis in the infected macrophages.

### 3.2 Caspase-1 and caspase-11 are required for the activation of GSDMD induced by *C. trachomatis*

**Figure 2:**
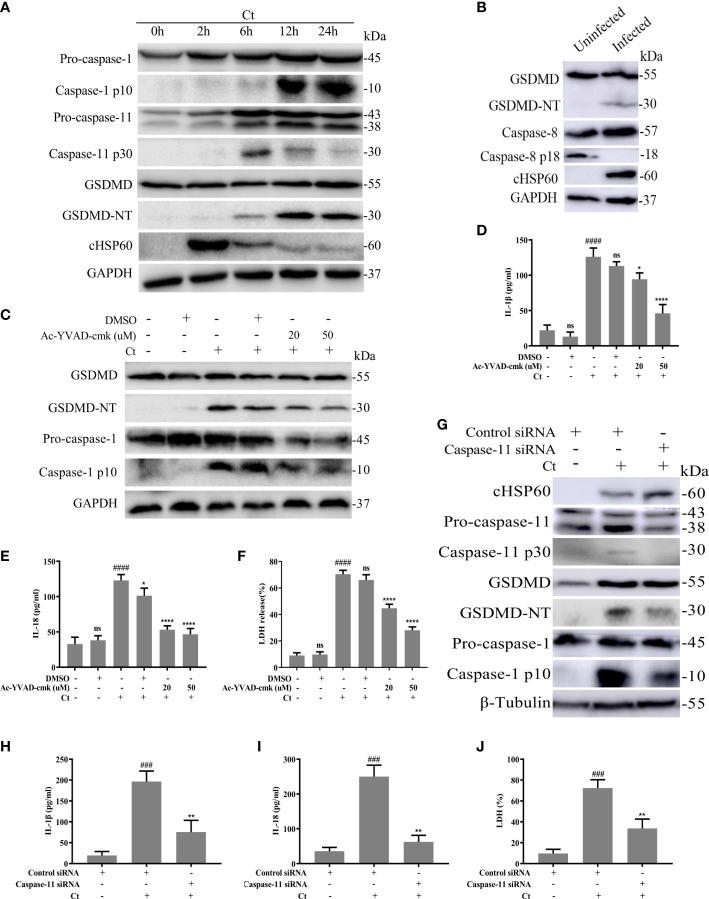
Chlamydia-induced GSDMD activation is accompanied by activation of Caspase-1 and caspase-11. **(A)** Immunoblot analysis showed expression of caspase-1, caspase-11, GSDMD and cHSP60 in RAW264.7 cells with *C*. *trachomatis* infection (MOI=5) at different time points. **(B)** Immunoblot analysis showed expression of GSDMD, caspase-8 and cHSP60 in RAW264.7 cells with *C*. *trachomatis* infection (MOI=5) at 12 hours post infection. For **(C–F)**, RAW264.7 cells were pretreated with Ac-YVAD-cmk for 30 minutes, then infected with *C*. *trachomatis* (MOI=5) for 12 hours. The same concentration of Ac-YVAD-cmk was maintained throughout the rest of the infection. **(C)**. Immunoblot analysis showed expression of GSDMD and caspase-1 in cell extract in RAW264.7 cells. For **(G–J)**, RAW264.7 cells were transfected with negative control siRNA or caspase-11 siRNA for 48 hours, then infected with *C*. *trachomatis* (MOI=5) for 12 hours. **(G)**. Immunoblot analysis showed expression of cHSP60, caspase-11, GSDMD and caspase-1 in cell extract in RAW264.7 cells. **(D, E, H, I)**. ELISA analysis showed IL-1β and IL-18 secretion in culture supernatant in RAW264.7 cells. **(F, J)**. LDH release in supernatant was measured to estimate cell death. ^###^P<0.001, ^####^P<0.0001 vs the control group, *P<0.05, **P<0.01, ****P<0.0001, vs the Ct group. ns represents no significance. n=3.

Caspase-1, caspase-11 or/and caspase-8 activations could regulate GSDMD-dependent pyroptosis (Gram et al., 2019). Here, we tried to explore whether GSDMD-mediated pyroptosis induced by *C. trachomatis* infection in macrophages was also affected by these activations. RAW264.7 cells were infected with *C. trachomatis* at a MOI of 5 and then cultured for different time course. We found caspase-1 was activated in a time-dependent manner in *C. trachomatis-*infected RAW264.7 cells (**Figure 2A**; **Figure S1A**). Furthermore, we observed that *C. trachomatis* also negatively regulated caspase-11 activation in a time-dependent manner within 24 hours (**Figure 2A**; **Figure S1B**). GSDMD was progressively activated with the ascending infection time and the greatest activation was observed at 12 hours post infection (**Figure 2A**; **Figure S1C**). We also found decreased expression of chlamydial heat shock protein 60 (cHSP60), the chlamydial marker protein, by prolonging infection time (**Figure 2A**; **Figure S1D**), indicating that macrophages could gradually inhibit Chlamydia replication. In addition, at 12 hours post infection, cleavage fragments of caspase-8 (p18) was not detected in the infected group compared with the uninfected group, indicating that caspse-8 was not involved in the cleavage of GSDMD (**Figure 2B**).These results suggested that caspase-1 and caspase-11 may play an important role in *C. trachomatis* clearance mediated by GSDMD activation.

To further evaluate the contributions of caspase-1 in GSDMD-mediated pyroptosis, RAW264.7 cells were infected with *C. trachomatis* at a MOI of 5, then cultured for 12 hours. We blocked the activations of caspase-1 with the specific inhibitor Ac-YVAD-cmk. Ac-YVAD-cmk treatment significantly reduced GSDMD-NT levels (**Figure 2C**; **Figure S2A**), proinflammatory cytokines IL-1β (**Figure 2D**) and IL-18 (**Figure 2E**) secretions, and LDH release (**Figure 2F**). These results indicated that caspase-1 could mediate GSDMD activation. To ascertain the role of caspase-11, RAW264.7 cells were transfected with control siRNA or caspase-11 siRNA and then were infected with *C. trachomatis* at a MOI of 5 for 12 hours. siRNA knockdown of caspase-11 strongly prevented GSDMD cleavage (**Figure 2G**; **Figure S3D**), reduced IL-1β (**Figure 2H**) and IL-18 (**Figure 2I**) secretions and LDH release (**Figure 2J**), which indicated that caspase-11 also mediated GSDMD activation. To our surprise, caspase-1 activation was inhibited upon caspase-11 knockdown (**Figure 2G**; **Figure S3E**). Inhibition of caspase-11 expression with a caspsae-11 inhibitor wedelolactone showed the same results (**Figure S4A**). Collectively, these data suggested that GSDMD-mediated pyroptosis and proinflammatory cytokines IL-1β and IL-18 release in *C. trachomatis*-infected macrophages could be stimulated *via* activating caspase-1 and caspase-11.

### 3.3 Blockade of GSDMD-dependent pyroptosis increases intracellular growth of *C. trachomatis* in macrophages

**Figure 3:**
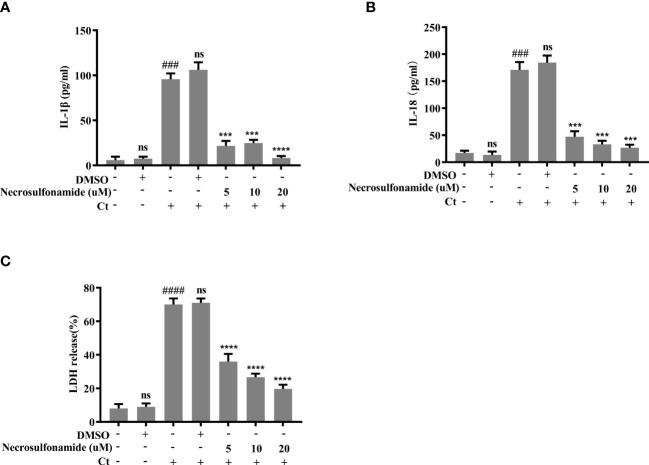
Chlamydia-induced pyroptosis is dependent on the activation of GSDMD. For **(A–C)**, RAW264.7 cells were pretreated with Necrosulfonamide for 30 minutes, then infected with *C. trachomatis* (MOI=5) for 12 hours. Same concentration of Necrosulfonamide was maintained throughout the rest of the infection. **(A, B)**. ELISA analysis showed IL-1β and IL-18 secretion in culture supernatant in RAW264.7 cells. **(C)**. LDH release in supernatant was measured to estimate cell death. ^###^P<0.001, ^####^P<0.0001 vs the control group, ***P<0.001, ****P<0.0001, vs the Ct group. ns represents no significance. n=3.

**Figure 4:**
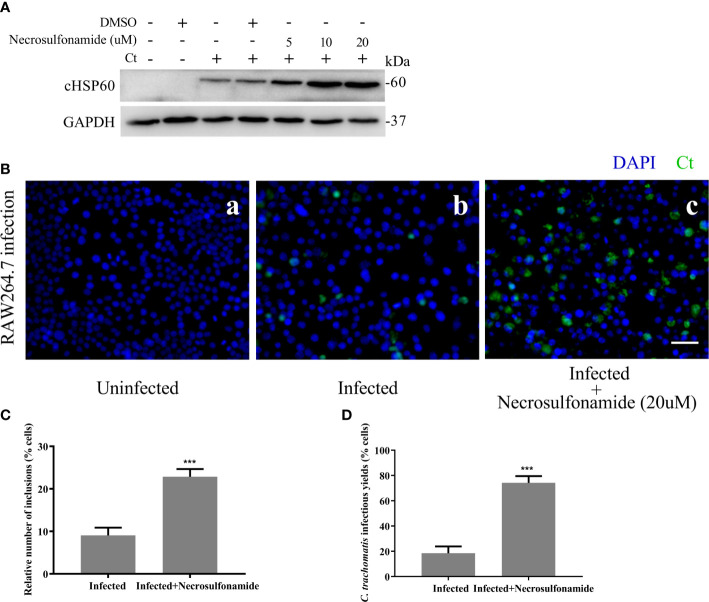
Blocking GSDMD pore-forming activity significantly increases the intracellular growth of *C*. *trachomatis.* **(A)** RAW264.7 cells were pretreated with Necrosulfonamide for 30 minutes, then infected with *C*. *trachomatis* (MOI=5) for 12 hours. The same concentration of Necrosulfonamide was maintained throughout the rest of the infection. Immunoblot analysis showed expression of cHSP60 in cell extract in RAW264.7 cells. **(B, C)**. RAW264.7 cells pretreated with Necrosulfonamide for 30 minutes, then infected with *C*. *trachomatis* (MOI=5) for 40 hours. Same concentration of Necrosulfonamide was maintained throughout the rest of the infection. **(B)**. Immunofluorescence microscopy analysis showed the growth of *C*. *trachomatis* in RAW264.7 cells. **(C)**. Quantitative analysis of inclusion number relative to nuclei in RAW264.7cells in **(B)**. **(D)** RAW264.7 cells were lysed to collect *C*. *trachomatis* infectious progenies to re-infect HeLa cells for 24 hours, and immunofluorescence microscopy quantitative analysis of inclusion number relative to nuclei in HeLa cells was used to estimate *C. trachomatis* infectious yields within RAW264.7 cells. Scale bars, 20 μm. ***P<0.001, vs the Infected group. n=3.

We next sought to determine the effect of GSDMD-mediated pyroptosis on chlamydia survival in macrophages. Necrosulfonamide, a potent specific inhibitor of GSDMD, which could restrain the oligomerization of GSDMD-NT and pyroptotic pore formation (Rathkey et al., 2018), was used in this study. RAW264.7 cells were infected with *C. trachomatis* at a MOI of 5. After Necrosulfonamide treatment, proinflammatory cytokines IL-1β (**Figure 3A**) and IL-18 (**Figure 3B**) secretions and LDH release (**Figure 3C**) in *C. trachomatis*-infected RAW264.7 cells at 12 hours post infction were decreased in a dose*-*dependent manner. These results indicated that Necrosulfonamide could inhibit pyroptosis in macrophages *via* blocking the pores formation in the cell membrane. It is worth noting that the expression levels of cHSP60 positively correlated with increased concentrations of necrosulfonamide in the RAW264.7 cells (**Figure 4A**). This result suggested that pyroptosis could restrict chlamydial growth in macrophages. Immunofluorescence assays were conducted at 40 hours post infection for further verification. As expected, in the presence of Necrosulfonamide, the number and the size of *C. trachomatis* inclusions were significantly increased in RAW264.7 cells compared to untreated group (**Figures 4B, C**). Infectious *C. trachomatis* progenies in RAW264.7 cells went up in the re-infection assay in HeLa cells (**Figure 4D**). Therefore, GSDMD-mediated pyroptosis is critical for limiting *C. trachomatis* growth in macrophages.

In summary, we proposed that GSDMD-mediated pyroptosis could be a defensive mechanism against *C. trachomatis* infection in macrophages through cleaving executor GSDMD to GSDMD-NT with pore-forming activity. The latter formed pyroptotic pores in cell membrane, consequently led to cell lytic death and proinflammatory cytokines secretion.

### 3.4 Pyroptosis mediated by Caspase-1 and -11-activated GSDMD is able to inhibit the intracellular growth of *C. trachomatis*

**Figure 5:**
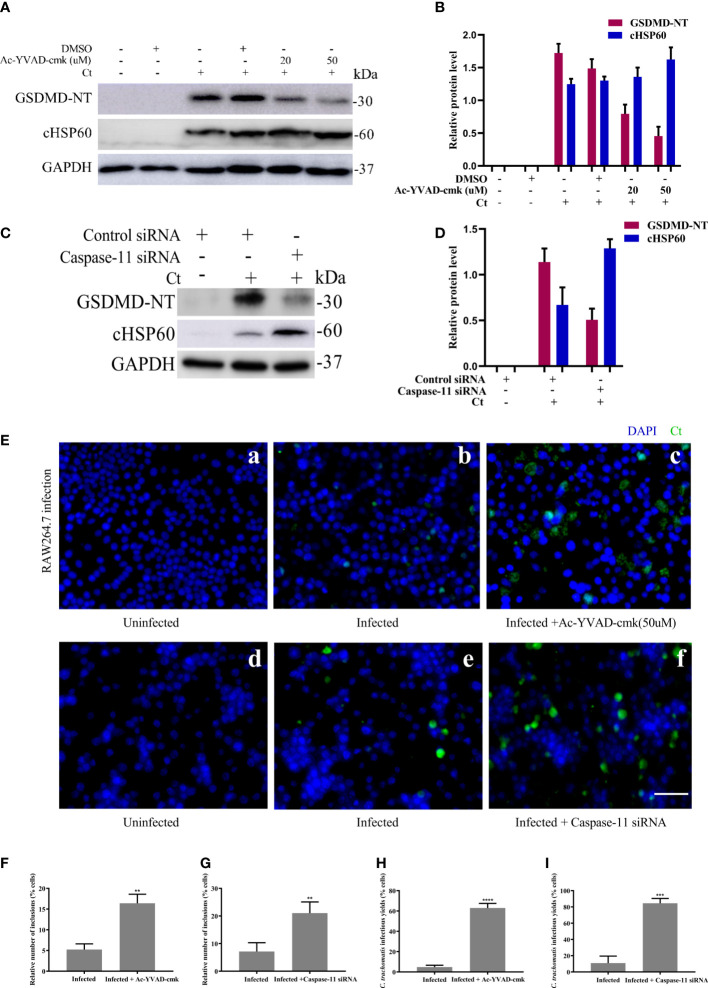
Inhibition of caspase-1 and caspase-11 activation increases *C*. *trachomatis* growth in macrophages. **(A)**. RAW264.7 cells were pretreated with Ac-YVAD-cmk for 30 minutes, then infected with *C*. *trachomatis* (MOI=5) for 12 hours. Same concentration of Ac-YVAD-cmk was maintained throughout the rest of the infection. Immunoblot analysis showed expression of GSDMD and cHSP60 in RAW264.7 cells. **(B)**. The quantitative analysis of GSDMD and cHSP60 protein levels relative to GAPDH protein levels in **(A)**. **(C)**. RAW264.7 cells were transfected with negative control siRNA or caspase-11 siRNA for 48 hours, then infected with *C*. *trachomatis* (MOI=5) for 12 hours. Immunoblot analysis showed expression of GSDMD and cHSP60 in RAW264.7 cells. **(D)**. The quantitative analysis of GSDMD and cHSP60 protein levels relative to GAPDH protein levels in **(C)**. **(E)** RAW264.7 cells pretreated with Ac-YVAD-cmk for 30 minutes, then infected with *C. trachomatis* (MOI=5) for 40 hours, same concentration of Ac-YVAD-cmk was maintained throughout the rest of the infection. In addition, RAW264.7 cells were transfected with negative control siRNA or caspase-11 siRNA for 48 hours, then infected with *C. trachomatis* (MOI=5) for 12 hours. Immunofluorescence microscopy analysis showed the growth of *C*. *trachomatis* in RAW264.7 cells. **(F, G)**. Quantitative analysis of inclusion number relative to nuclei in RAW264.7cells in **(E)**. **(H, I)**. RAW264.7 cells in **(E)** were lysed to collect *C. trachomatis* infectious progenies to re-infect HeLa cells for 24 hours, and immunofluorescence microscopy quantitative analysis of inclusion number relative to nuclei in HeLa cells was used to estimate *C. trachomatis* infectious yields within RAW264.7 cells. Scale bars, 20 μm. **P<0.01, ***P<0.001, ****P<0.0001, vs the Infected group. n=3.

Finally, we suppressed the activation of upstream molecules caspase-1 and caspase-11 to further study the role of pyroptosis. RAW264.7 cells were infected with *C. trachomatis* at a MOI of 5 for 12 and 40 hours. With Ac-YVAD-cmk treatment, RAW264.7 cells presented an increased *C. trachomatis* loads (**Figure 5A**) and inclusion numbers (**Figures 5E, F**). Similarly, there were rising *C. trachomatis* loads (**Figure 5C**) and the inclusion numbers (**Figures 5E, G**) upon caspase-11 knockdown. We also verified the requirements of caspase-1 and caspase-11 activations in RAW264.7 cells for restricting infectious *C. trachomatis* progeny production by reinfection assay. *C. trachomatis* infected-RAW264.7 cells were lysed at 40 hours post infection and then the infectious *C. trachomatis* progenies within cells were harvested and used to re-infect HeLa cells. With Ac-YVAD-cmk treatment or upon caspase-11 knockdown, there was an obvious increase of infectious *C. trachomatis* yield*s* in RAW264.7 cells as supported by the increasing inclusion numbers in the HeLa cells (**Figures 5H, I**). Thus, these results indicated that both caspase-1 or caspase-11 contributed to restraining chlamydial growth in macrophages. Notably, GSDMD-NT levels decreased when RAW264.7 cells were treated with Ac-YVAD-cmk treatment or transfected by caspase-11 siRNA, while cHSP60 expression levels increased with the progressive inhibition of GSDMD (**Figures 5A–D**). Therefore, inactivation of caspase-1 or caspase-11 inhibited GSDMD-mediated pyroptosis, which promoted the survival of *C. trachomatis*. In conclusion, these data suggested that pyroptosis mediated by caspase-1 and -11-activated GSDMD is able to inhibit the intracellular growth of *C. trachomatis*.

## 4 Discussion

In this study, we characterized the role of GSDMD-mediated pyroptosis during *C. trachomatis* infection in macrophages. Firstly, we clearly demonstrated that the pyroptosis occurred in macrophages with *C. trachomatis* infection. Additionally, evidence was provided showing that caspase-1 and caspase-11 activation were required for GSDMD-mediated pyroptosis. Specifically, our study revealed that caspase-1/11-GSDMD-mediated pyroptosis may lead to cell lytic death and proinflammatory cytokines secretion, which restrained *C. trachomatis* growth in macrophages.

Our study found that inhibition of *C. trachomatis* growth was dependent on GSDMD-NT pore-forming activity. GSDMD-NT can form extensive pores on phosphoinositide- or cardiolipin-containing liposomes or on liposomes made of natural polar lipid mixtures (Shi et al., 2017), some essential constituents of chlamydial inclusion membrane (Yao et al., 2015). Thus, GSDMD-NT may exhibit strong membrane pore-forming activity on both the cell plasma membrane and chlamydial inclusion membrane. In fact, GSDMD-NT can form pores not only in the plasma membrane, but also in bacterial membranes (Jorgensen et al., 2016), mitochondrial membranes (Estfanous et al., 2021) and phagosomal membranes (Estfanous et al., 2021). Hence, apart from forming pores in the cell membrane, we speculate that GSDMD-NT may also form pores in inclusion membrane directly, resulting in destruction of chlamydial reproductive niche and contributing to *C. trachomatis* clearance although this hypothesis requires more experimental evidence to confirm.

GSDMD-mediated infection clearance was demonstrated in several intracellular bacteria. Pyroptotic cell death deprives intracellular pathogens from their replicative niches as a cell-intrinsic mechanism to control infection (Jorgensen and Miao, 2015). Chlamydiae are obligately intracellular pathogens, it has been extensively documented the premature death of *C. trachomatis*-infected cells disrupts the ability of the bacteria to form infectious EBs. Hence, we focus on whether pyroptosis can lead to cell-intrinsic inhibition of *C. trachomatis*. We observed *C. trachomatis* infection induced-pyroptosis occurred most strongly at early infection as the greatest activation of GSDMD-NT was observed at 12 hours post infection (**Figure 2A**). In this period, EB differentiates into RB and the rupture of pyroptotic cells deprives the replicative niche required for RBs. As a result, RB replication is diminished, disallowing the RBs to differentiate back into the infectious EBs. The combined outcome of these effects is cell-intrinsic inhibition of *C. trachomatis*. This is consistent with previous studies showing that *C. trachomatis*-infected mouse macrophages undergo pyroptosis early in infection (Finethy et al., 2015; Webster et al., 2017).

Caspase-11 directly acts as a receptor of cytosolic bacterial lipopolysaccharide (LPS) and is activated by binding to LPS (Shi et al., 2014). Our results showed that *C. trachomatis* organisms activated caspase-11 in macrophages. Paradoxically, a recent study has shown that *C. trachomatis* LPS could not activate caspase-11, as shown by the lack of GSDMD activation in murine bone marrow-derived macrophages (BMDMs) transfected with Chlamydia LPS (Yang et al., 2019). But several studies found that Chlamydia infection did induce caspase-11 activation (Finethy et al., 2015; Webster et al., 2017). This may be caused by differences in the ability of Chlamydia LPS and *C. trachomatis* organism activating caspase-11. Webster et al. have reported that caspase-11 expression was markedly reduced in BMDMs stimulated with irradiated *C. trachomatis*, suggesting that caspase-11 expression requires additional, essential signals provided by *C. trachomatis* replication and/or metabolism intracellularly. NF-κB activation is essential for caspase-11 expression in response to LPS (Schauvliege et al., 2002).Whereas transfection of Chlamydia LPS into macrophages did not activate NF-κB (Yang et al., 2019). Our experiments indicated that *C. trachomatis* organisms can activate NF-κB (data not shown). Thus, the fact that *C. trachomatis* organisms are capable of activating caspase-11 in macrophages is not contradictory. However, the exact mechanisms of caspase-11 activation remain to be elucidated.

*C. trachomatis* infection results in caspase-1 activation *via* activation of NLRP3 and AIM2 inflammasome. NLRC4 and NLRP1, which activate caspase-1 without ASC, were not activated based on evidence in the literature using BMDMs (Finethy et al., 2015). We found that *C. trachomatis* infection activated caspase-1 using immortalized mouse bone marrow-derived macrophages (iBMDMs) expressing ASC protein (**Figure S5A**), which was consist with previous study (Finethy et al., 2015). Surprisingly, caspase-1 was activated in RAW264.7 cells deficient in ASC acquired for NLRP3 and AIM2 in this study. This observation appeared to be inconsistent with previous research. A possible explanation for this apparent contradiction is that there may be a caspase-1 activation pathway independent of NLRP3, AIM2, NLRC4, and NLRP1 activation in RAW264.7 cells infected with *C. trachomatis*, which is supported by the two studies. One study reported that caspase-1 was activated treated with nigerin, a NLRP3 classic activator, in RAW26.7 cells, suggesting the existence of a NLRP3 inflammasome-independent caspase-1 activation pathway (Armstrong et al., 2019). Another study also demonstrated that caspase-1 was activated through an inflammasome-independent pathway in RAW264.7 cell model (Chuang et al., 2020). Therefore, *C. trachomatis* is likely to activate caspase-1 through an inflammasome- independent pathway in RAW264.7 cells. The underlying mechanism for regulating caspase-1 activation will be a challenging but interesting future research direction.

We have noticed that caspase-11 knockdown had a strong protection from cell death (**Figures 2H–J**) and promoted Chlamydia survival (**Figures 5C, E, G**) since caspase-11 knockdown significantly inhibited pyroptosis. Caspase-11 knockdown not only inhibited GSDMD activation, but also inhibited caspase-1 activation unexpectedly (**Figure 2G**). It is well-established in the literature that caspase-11 activation leads to non-canonical NLRP3 inflammasome assembly and caspase-1 activation (Kayagaki et al., 2011). By surprise, caspase-11 knockdown inhibited caspase-1 activation significantly in NLRP3 inflammasome-deficient Raw264.7 cells (**Figure 2G**). How caspase-11 regulates a NLRP3 inflammasome-independent caspase-1 activation pathway needs further exploration. Moreover, caspase-11 inhibitor wedelolacetone treatment produced the same effect as caspase-11 siRNA transfection shown in **Figure 2G**. Despite being widely available, wedelolacetone inhibits caspase-11 expression indirectly. Wedelolactone has been reported to inhibit caspase-11 expression by inhibiting the NF-κB pathway since caspase-11 gene expression in response to LPS requires NF-κB activation (Schauvliege et al., 2002; Kobori et al., 2004). Although the possibilities of NF-κB signaling inhibition suppressing pyroptosis cannot be ruled out, wedelolacetone treatment inhibited caspase-11 expression and blocked its activation (**Figure S4A**) and these results are consistent with results from caspase-11 knockdown (**Figure 2G**), which indicated that these observed phenotypes may be at least partially attributable to the inhibitory effect of wedelolacetone on caspase-11 expression. In addition, our result showed that necrosulfonamide treatment largely prevented GSDMD-NT pore formation (**Figure 3**). The off-target effects of necrosulfonamide in this study were less likely. Because necrosulfonamide, a known inhibitor of MLKL, has no effect on necrosis in murine cells (Sun et al., 2012).

Some researchers believed that macrophages are permissive for *C. trachomatis* growth. However, the growth in macrophages is severely limited compared to that observed in epithelial cells (Beagley et al., 2009). Macrophages could restrict intracellular growth of *C. trachomatis* through distinct mechanisms including lysosomal degradation (Kuo et al., 2002; Kinchen and Ravichandran, 2008; Yasir et al., 2011), autophagy (Al-Zeer et al., 2009; Al-Zeer et al., 2013) and nutrient starvation (Heuer et al., 2009; Soliman et al., 2010; Luo et al., 2019). Previous research has inferred that pyroptosis is detrimental to Chlamydia infection. By inhibiting GSDMD-mediated pyroptosis, we found that the infectious yield of *C. trachomatis* are increased in RAW264.7 cells (**Figure 4D**). Thus, our study further supported the idea that pyroptosis is indeed a host defense mechanism against Chlamydia infection and may help explain why the macrophage environment is hostile to chlamydial growth.

**Figure 6:**
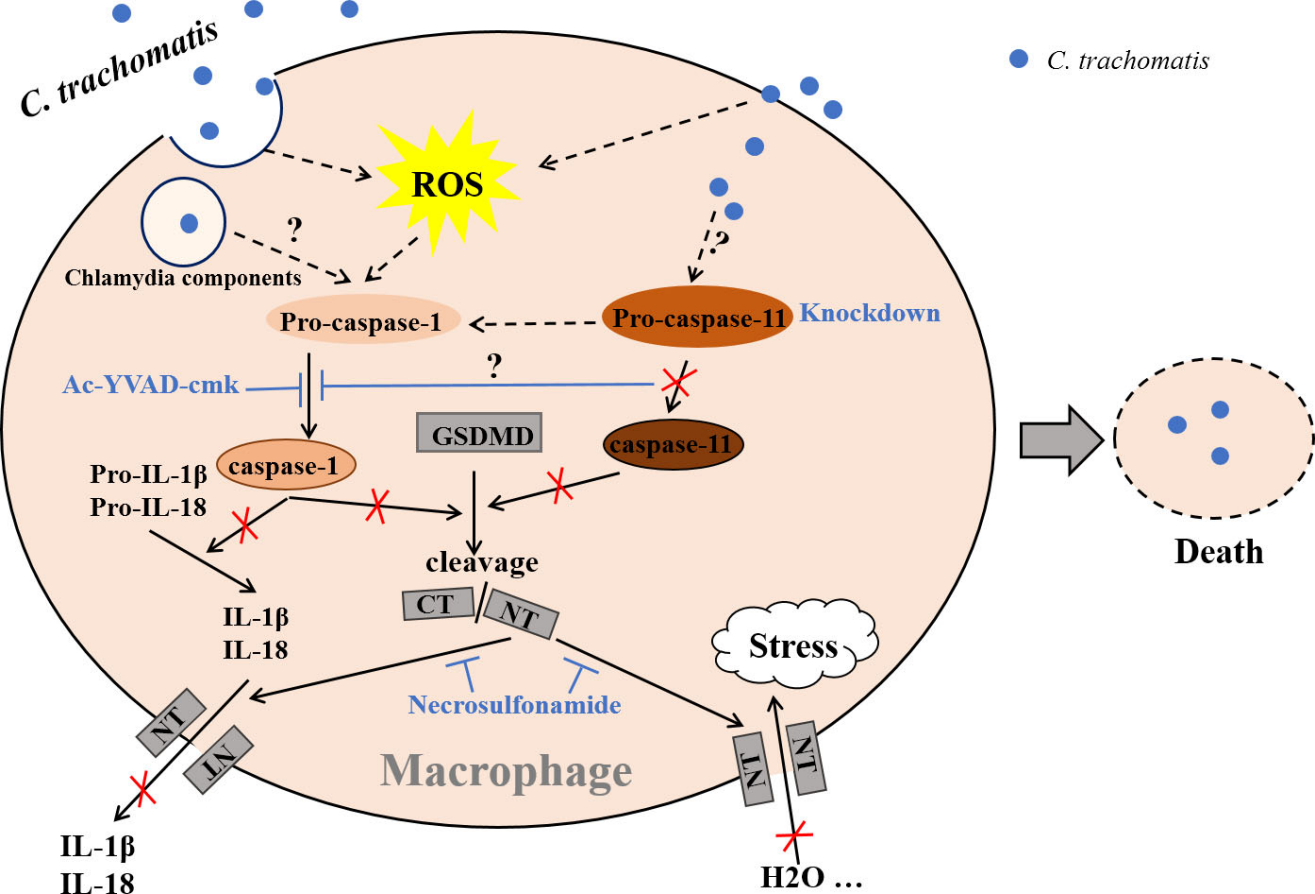
Proposed model of chlamydial growth restriction in macrophages by GSDMD-mediated pyroptosis. *C. trachomatis* is internalized by macrophages after contacting them during infecting host, cleaving GSDMD into GSDMD C-terminus (GSDMD-CT) and GSDMD-NT *via* activating caspase-1/11. GSDMD-NT could insert itself into the cell membrane to form pores. H2O influx leads to an increase in intracellular osmotic pressure, then causing cell swelling and rupture, destroying the replication niche of *C. trachomatis* to restrict its growth in macrophages. Active caspase-1 cleaves Pro-IL-1β and Pro-IL-18 into mature IL-1β and IL-18. Both are then released into the outside of cells during pyroptosis. Caspase-1 activation inhibition with Ac-YVAD-cmk blocks IL-1β and IL-18 mature and GSDMD cleavage. Caspase-11 knockdown inhibits both GSDMD activation and caspase-1 activation. Necrosulfonamide restrains treatment GSDMD-NT oligomerization and pyroptotic pore formation, inhibiting IL-1β and IL-18 secretion and H2O influx. Dashed lines represent published pathways, solid lines represent pathways described in this study. It is currently unknown what *C. trachomatis* components activate caspase-1 or caspase-11(not by Chlamydia LPS). It is unclear how caspase-11 signal pathway interacts with caspase-1 signal pathway.

In summary, we presented here a model of intracellular chlamydial growth inhibition in macrophages (**Figure 6**). Caspase-1 and caspase-11 are activated in *C. trachomatis*-infected macrophages to cleave GSDMD into GSDMD-NT, causing pyroptotic cell lysis and IL-1β and IL-18 secretions, which limited *C. trachomatis* growth. The *C. trachomatis*-mouse macrophage interaction model may be used to further investigate how *C. trachomatis* triggers caspase-1/11 activation and how *C. trachomatis* is inhibited in the pyroptotic cells. This study is helpful to understand the pathophysiological process of Chlamydia infection and reveals novel targets for treating *C. trachomatis* infection.

## Data availability statement

The raw data supporting the conclusions of this article will be made available by the authors, without undue reservation.

## Author contributions

PJ, HC, NZ, JC, and LT designed the experiments. PJ, HC, XF, MJ, DX, HT, and LZ performed the experiments. PJ, XF, and HX analyzed data and wrote the manuscript. HX, NZ and LT revised the manuscript. All authors contributed to the article and approved the submitted version.
