# Supplementary material for: GSDMD-mediated pyroptosis restrains intracellular Chlamydia trachomatis growth in macrophages
Source: Front Cell Infect Microbiol. 2023 Mar 16;13:1116335. doi: 10.3389/fcimb.2023.1116335 (PMC10061094; doi:10.3389/fcimb.2023.1116335)
Supplement: Supplementary file 1 [file DataSheet_1.docx]

Supplementary Material

**Supplementary Figures**

**
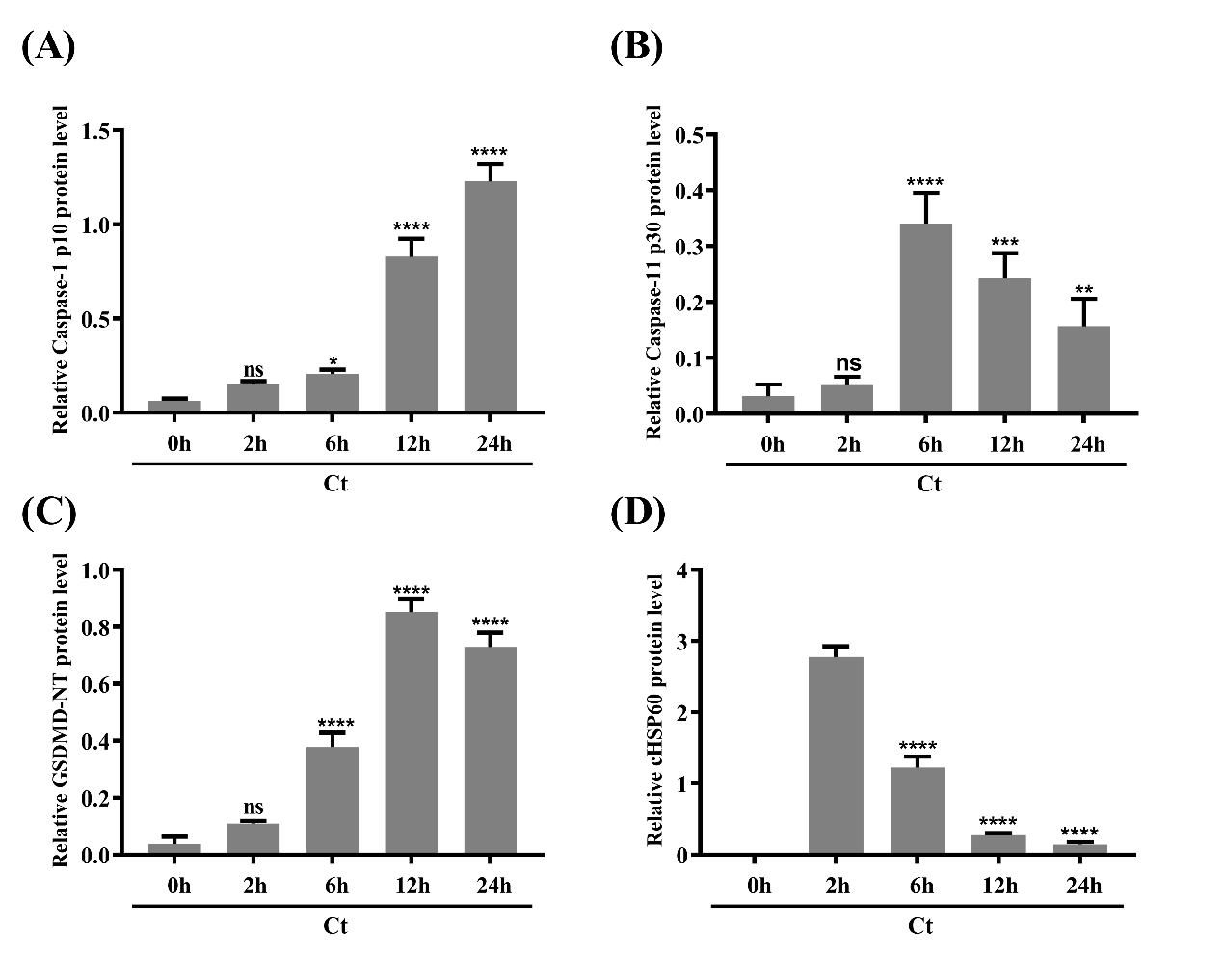
**

**Figure S1.** **Quantification of the Western blot data in Figure 2A**

(**A**) The quantification of Caspase-1 p10 band was normalized first to GAPDH then to the Pro-caspase-1 band intensity. (**B**) The quantification of Caspase-11 p30 band was normalized first to GAPDH then to the Pro-caspase-11 band intensity. (**C**) The quantification of GSDMD-NT band was normalized first to GAPDH then to the GSDMD band intensity. *P˂0.05, ***P˂0.001, ****P˂0.0001, vs the 0h group. ns represents no significance. n=3. (**D**) The quantification of cHSP60 band was normalized to GAPDH. ****P˂0.0001, vs the 2h group. ns represents no significance. n=3.


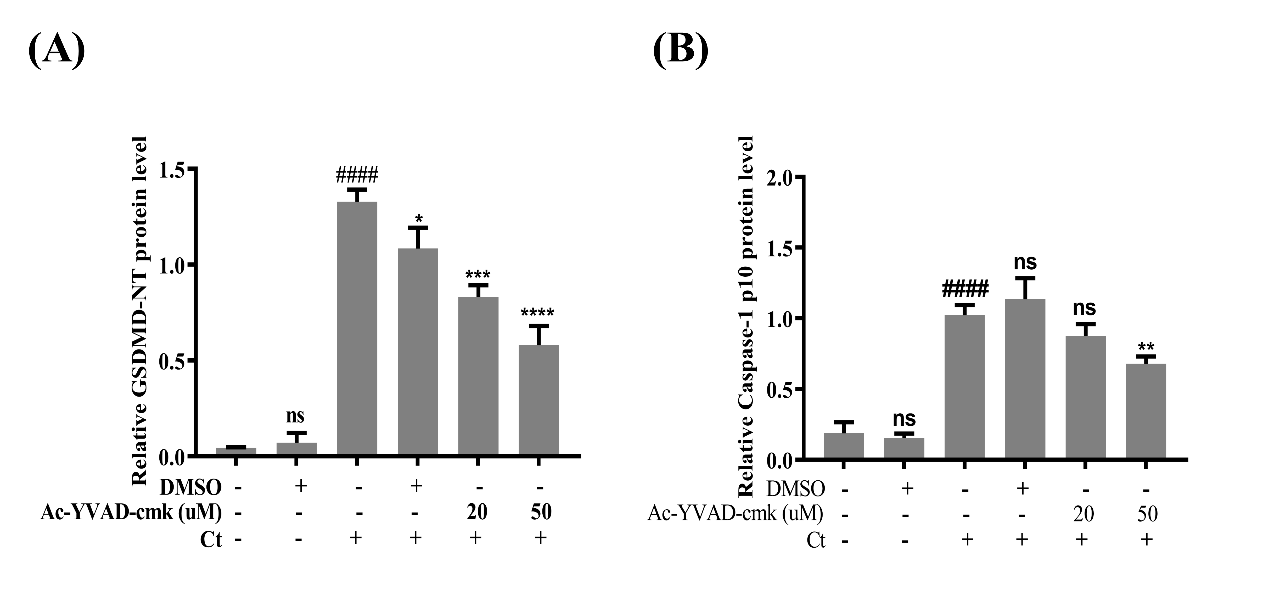


**Figure S2. Quantification of the Western blot data in Figure 2C**

(**A**) The quantification of GSDMD-NT band was normalized first to GAPDH then to the GSDMD band intensity. (**B**) The quantification of Caspase-1 p10 band was normalized first to GAPDH then to the Pro-caspase-1 band intensity. ^####^P˂0.0001 vs the control group, *P˂0.05, **P˂0.01, ***P˂0.001, ****P˂0.0001, vs the Ct group. ns represents no significance. n=3.


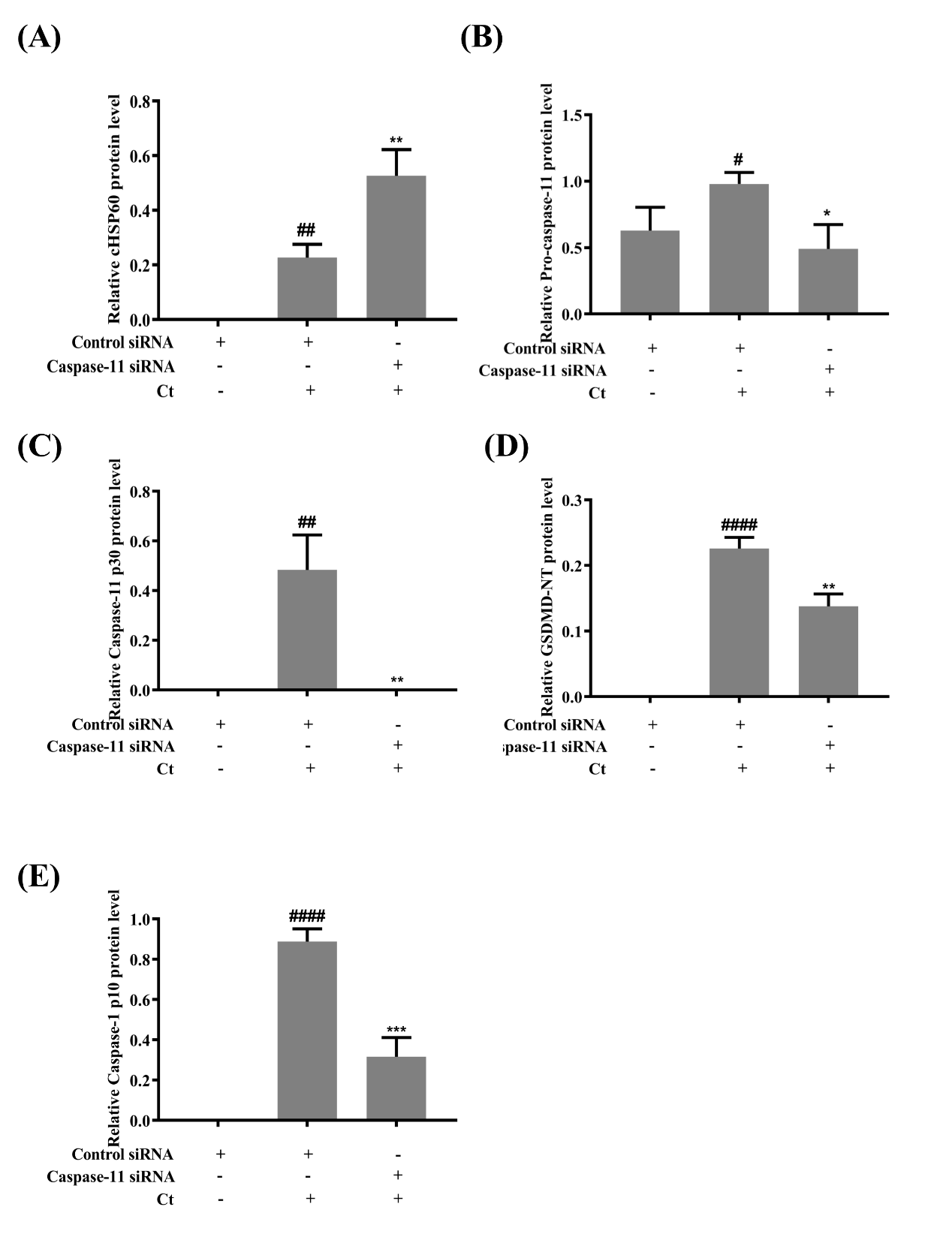


**Figure S3. Quantification of the Western blot data in Figure 2G**

(**A**) The quantification cHSP60 band was normalized to GAPDH band intensity. (**B**) The quantification Pro-caspase-11 band was normalized to GAPDH band intensity. (**C**) The quantification of Caspase-11 p30 band was normalized first to GAPDH then to the Pro-caspase-11 band intensity. (**D**) The quantification of GSDMD-NT band was normalized first to GAPDH then to the GSDMD band intensity. (**E**) The quantification of Caspase-1 p10 band was normalized first to GAPDH then to the Pro-caspase-1 band intensity. ^#^P˂0.05, ^##^P˂0.01, ^####^P˂0.0001 vs the control group, **P˂0.01，*P˂0.05, ***P˂0.001, vs the Ct group. ns represents no significance. n=3.

**
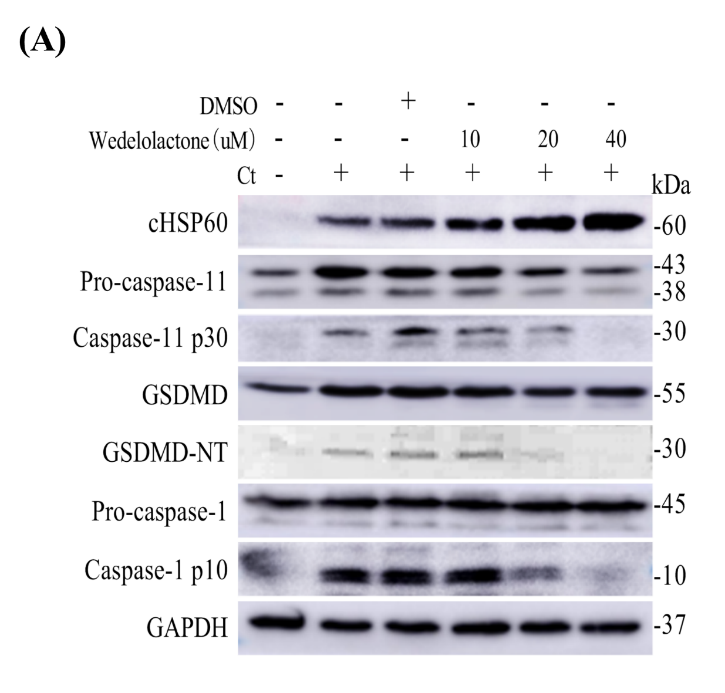
**

**Figure S4. Caspase-11 are required in *C. trachomatis*-induced GSDMD activation.**

RAW264.7 cells were pretreated with wedelolactone for 30minutes, then infected with *C. trachomatis* (MOI=5) for 12h. The same concentration of wedelolactone was maintained throughout the rest of the infection. **(A).** Immunoblot analysis showed expression of cHSP60, caspase-11, GSDMD and caspase-1 in cell extract in RAW264.7 cells.

**
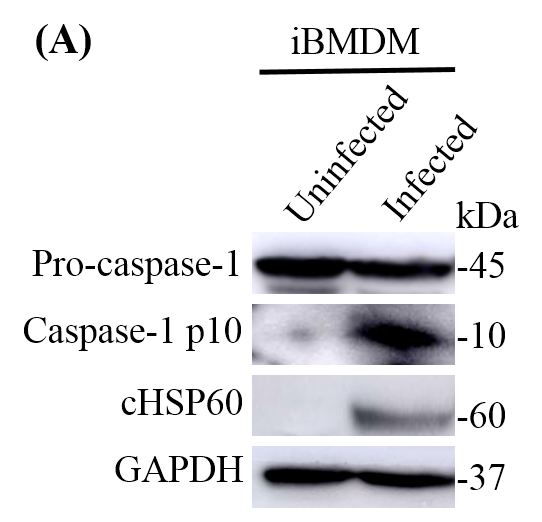
**

**Figure S5. Caspase-1 was activated in *C. trachomatis*-infected iBMDM cells.**

iBMDM cells were infected with *C*. *trachomatis* (MOI=5) for 12 hours. **(A)**. Immunoblot analysis showed caspase-1 activation in *C*. *trachomatis-*infected iBMDM cells. cHSP60 is as marker of *C. trachomatis* infection.
